# High-Intensity Interval Training for Early Post-Acute Myocardial Infarction - A Promising Approach for Rats, but what about Human Beings?

**DOI:** 10.5935/abc.20180068

**Published:** 2018-04

**Authors:** Ricardo Stein

**Affiliations:** Universidade Federal do Rio Grande do Sul, Porto Alegre, RS - Brazil

**Keywords:** Acute Coronary Syndrome, Cardiac Rehabilitation, Exercise

Acute coronary syndromes (ACSs), in particularly, acute myocardial infarction (AMI), kill or debilitate a large number of patients in the world. Despite the fact that not all patients develop ventricular dysfunction after the event, there is still a high prevalence of post-AMI heart failure,^1,2^ which is considered a public health problem. Although the management of post-ACS is based on a wide range of drugs, usually associated with the revascularization procedure, different non-pharmacological strategies have been shown useful. In this regard, physical exercise is indicated,^3^ including cardiac rehabilitation programs that usually combine aerobic and resistance training with stretching exercises.

Nevertheless, there is no single recipe for prescribing exercise after an acute coronary event. In my opinion, cardiologists should formally prescribe physical exercise in addition to cardiovascular drugs, considering aspects such as dosage, intervals, intensity and potential side effects. With respect to physical training, a vast of different exercise modalities have emerged and applied in health. Pilates, Tai Chi Chuan, functional training, crossfit, high-intensity interval training (HIIT) among others have spread across gyms and physical centers over the country and have been practiced primarily by apparently healthy individuals. As time passed, animal experiments and clinical studies on cardiovascular disease patients have been conducted.^4-7^ HIIT was first proposed to Japanese Olympic skaters by Izumi Tabata. Today, HIIT consists in sessions of one to four-minute of high-intensity submaximal load alternating with low-to-moderate intensity exercises. Randomized clinical trials involving small samples have suggested a superiority of the method in increasing peak oxygen uptake (VO_2_peak) as compared with conventional continuous training. Due to its peculiarities and results, HIIT has boomed all over the world; however, international literature showing the impact of the method in ischemic heart disease patients, particularly in post-AMI patients is still lacking.^4,8,9^

In this journal issue, Winter et al.^10^ report information on the effects of HIIT on functional capacity and ventricular function in 29 Wistar rats after AMI. On day 21 after the event, the animals were randomized to control group (n = 10), or to undergo continuous training (n = 9) or HIIT (n = 10). All animals had ejection fraction equal to or greater than 50%, i.e., without ventricular dysfunction. An important finding was that the authors did not find within- or between group differences in echocardiographic findings before and after training in the animals allocated to continuous training or to HIIT. The authors suggest that both methods can increase functional capacity without altering ventricular function (remodeling). Based on this, one may ask the following question: can patients at early stage after AMI, without ventricular dysfunction, undergo this type of physical training?

In a classical study by Wisloff et al.,^5^ the authors evaluated three groups of elderly patients with heart failure and reduced ejection fraction (HFrEF), who were clinically stable and had had a myocardial infarction more than one year before the study. Patients were randomized to control, moderate continuous training (MCT) or HIIT group.^5^ Individuals assigned to HIIT showed improved peak VO_2_, left ventricular remodeling and reduced natriuretic peptide (BNP) levels as compared with MCT. Also, a meta-analysis involving 160 patients showed that interval training (regardless of its intensity) increased peak VO_2_ in HFrEF patients.^11^ Similarly, in a meta-analysis including 230 patients, Elliott et al.^12^ reported that interval training seems to increase peak VO_2_ in patients with stable coronary artery disease.^12^

In the last years, different strategies that can be included in early post-acute rehabilitation programs have emerged, such as Tai Chi Chuan.^13^ In any case, all interventions that may improve patients’ recovery and functional capacity, and whenever possible, increase patients’ survival should be used. With respect to the applicability of HIIT in the management of early post-AMI patients, it may be speculated that the method is efficient in improving peak VO_2_, an important prognostic marker. In fact, in the world of coronary stents and since post-AMI myocardial function is preserved in many patients, HIIT may be an attractive training strategy for some patients. On the other hand, the body of scientific knowledge is not sufficiently consistent to definitely recommend HIIT as a training modality for early post-AMI patients. Anyway Winter et al.,^10^ in their investigation on laboratory animals, take an important step towards an effective alternative for cardiac rehabilitation programs in this group of patients.
